# Viral Pneumonitis Is Increased in Obese Patients during the First Wave of Pandemic A(H1N1) 2009 Virus

**DOI:** 10.1371/journal.pone.0055631

**Published:** 2013-02-13

**Authors:** Jen Kok, Christopher C. Blyth, Hong Foo, Michael J. Bailey, David V. Pilcher, Steven A. Webb, Ian M. Seppelt, Dominic E. Dwyer, Jonathan R. Iredell

**Affiliations:** 1 Centre for Infectious Diseases and Microbiology Laboratory Services, Institute of Clinical Pathology and Medical Research, Sydney Institute for Emerging Infections and Biosecurity, University of Sydney, Westmead Hospital, Westmead, New South Wales, Australia; 2 Centre for Research Excellence in Critical Infections, University of Sydney, Westmead Hospital, Westmead, New South Wales, Australia; 3 School of Paediatrics and Child Health, University of Western Australia, Perth, Western Australia, Australia; 4 Department of Paediatrics and Adolescent Medicine and PathWest Laboratory Medicine WA, Princess Margaret Hospital, Subiaco, Western Australia, Australia; 5 Department of Microbiology and Infectious Diseases, Sydney South West Pathology Service, Liverpool Hospital, Sydney, New South Wales, Australia; 6 Australian and New Zealand Intensive Care Research Centre, Department of Epidemiology and Preventive Medicine, School of Public Health and Preventive Medicine, Monash University, Melbourne, Victoria, Australia; 7 Australian and New Zealand Intensive Care Society Centre for Outcome and Resource Evaluation, Carlton, Victoria, Australia; 8 Department of Intensive Care Medicine, The Alfred Hospital, Phahran, Victoria, Australia; 9 Intensive Care Unit, Royal Perth Hospital, Perth, Western Australia, Australia; 10 School of Medicine and Pharmacology and School of Population Health, University of Western Australia, Perth, Western Australia, Australia; 11 Department of Intensive Care Medicine, Nepean Hospital, Penrith, New South Wales, Australia; 12 Sydney Medical School, University of Sydney, Sydney, New South Wales, Australia; University of Liverpool, United Kingdom

## Abstract

**Introduction:**

There is conflicting data as to whether obesity is an independent risk factor for mortality in severe pandemic (H1N1) 2009 influenza (A(H1N1)pdm09). It is postulated that excess inflammation and cytokine production in obese patients following severe influenza infection leads to viral pneumonitis and/or acute respiratory distress syndrome.

**Methods:**

Demographic, laboratory and clinical data prospectively collected from obese and non-obese patients admitted to nine adult Australian intensive care units (ICU) during the first A(H1N1)pdm09 wave, supplemented with retrospectively collected data, were compared.

**Results:**

Of 173 patients, 100 (57.8%), 73 (42.2%) and 23 (13.3%) had body mass index (BMI) <30 kg/m^2^, ≥30 kg/m^2^ (obese) and ≥40 kg/m^2^ (morbidly obese) respectively. Compared to non-obese patients, obese patients were younger (mean age 43.4 vs. 48.4 years, p = 0.035) and more likely to develop pneumonitis (61% vs. 44%, p = 0.029). Extracorporeal membrane oxygenation use was greater in morbidly obese compared to non-obese patients (17.4% vs. 4.7%, p = 0.04). Higher mortality rates were observed in non-obese compared to obese patients, but not after adjusting for severity of disease. C-reactive protein (CRP) levels and hospital length of stay (LOS) were similar. Amongst ICU survivors, obese patients had longer ICU LOS (median 11.9 vs. 6.8 days, p = 0.017). Similar trends were observed when only patients infected with A(H1N1)pdm09 were examined.

**Conclusions:**

Among patients admitted to ICU during the first wave of A(H1N1)pdm09, obese and morbidly obese patients with severe infection were more likely to develop pneumonitis compared to non-obese patients, but mortality rates were not increased. CRP is not an accurate marker of pneumonitis.

## Introduction

Critical care data from the first pandemic influenza wave of 2009 (hereafter A(H1N1)pdm09) in Australia and New Zealand revealed that 28.6% of patients admitted to intensive care units (ICUs) were obese (defined as a body-mass index [BMI; calculated as weight in kilograms divided by height in metres squared] >35 kg/m^2^) [1], an observation not noted previously in severe influenza infection [2], [3]. Post hoc analysis revealed that the rate of obesity (BMI ≥30 kg/m^2^) in ICU patients (44%) was much higher than in the general Australian and New Zealand adult population (23.7%–27.1%) [4], [5].

This is comparable to data from the United States of America (USA), where 51% of patients hospitalized during the first pandemic phase in California had BMI ≥30 kg/m^2^, 2.2 and 1.5 times the prevalence of obesity in California and the USA respectively [6]. Of A(H1N1)pdm09-related deaths (overall mortality rate was 17%), 61% had BMI ≥30 kg/m^2^ and 30% had BMI ≥40 kg/m^2^.

Animal models have shown that obesity is associated with altered immune and inflammatory responses upon infectious stimuli [7]. Compared to lean mice, influenza infection in obese mice deregulates immune responses, and results in mortality rates six times higher [8]. The effect of the adipokines adiponectin and leptin, and cytokines on inflammatory cells (T-cells and monocytes/macrophages) in the obese population infected with severe influenza during a pandemic, is less well studied.

Obesity *per se* results in a chronic state of low-grade systemic inflammation, mediated by excess adipocyte production of pro-inflammatory cytokines including interleukin-6 (IL-6), tumour necrosis factor-α (TNF-α), monocyte chemoattractant protein-1 (MCP-1) and interleukin-8 (IL-8) [9], [10], [11]. Although anti-inflammatory cytokines are also produced, the net effect is a pro-inflammatory state [11]. Overweight and obese individuals also have high levels of circulating C-reactive protein (CRP) [9], [12], which may serve as a surrogate marker for IL-6, as production of CRP is stimulated by IL-6 [13].

An aberrant cytokine response (cytokine “storm”) in influenza infection may lead to viral pneumonitis and/or acute respiratory distress syndrome (ARDS) [14], which are postulated to be responsible for respiratory failure in obese patients with severe A(H1N1)pdm09 infection [15]. Although there may be multiple factors to account for poor outcomes in critically ill obese patients (including multiple co-morbidities, difficult and prolonged mechanical ventilation, increased nosocomial infections and other complications [16]), it has been suggested that obesity is an independent risk factor for death in severe A(H1N1)pdm09 infection [6], [17], [18], [19], [20].

This study aims to describe the demographic characteristics and outcomes of obese patients with severe influenza infection during the first A(H1N1)pdm09 wave in Australia; and to determine the relationship between BMI (as a marker of obesity), CRP and viral pneumonitis in severe influenza infection.

## Methods

The Australian and New Zealand Intensive Care Society (ANZIC) Influenza Investigators prospectively collected demographic, clinical and laboratory data from 722 patients admitted to 187 Australian and New Zealand adult and pediatric ICUs during the first A(H1N1)pdm09 pandemic wave from June 1^st^ to August 31^st^, 2009 [1]. The ANZICS Centre for Outcome and Resource Evaluation (ANZICS-CORE) collects data including postcodes, severity scores and associated biochemical and physiological values from the first 24 hours of ICU admission on patients admitted to 85% of ICUs in Australia and New Zealand. All patients defined as having ARDS or viral pneumonitis by the treating physician were grouped together as “pneumonitis”.

ANZIC and ANZICS-CORE data from adult patients admitted to nine ICUs in two Australian states, New South Wales and Western Australia were linked, and supplemented with CRP data (measured on admission to hospital and ICU) retrospectively collected from each hospital’s laboratory information system. The SEIFA (socioeconomic indexes for areas) index of disadvantages (available from http://www.abs.gov.au/AUSSTATS/abs@.nsf/DetailsPage/2033.0.55.0012006?OpenDocument), which summarizes various attributes (including income, unemployment, education attainment) and median household income (available from http://www.censusdata.abs.gov.au/censusOutput/abs@cpp2006.nsf) for each patient’s residential postal area was identified using the most recent census data (collected in 2006) from the Australian Bureau of Statistics. SEIFA is comprised of a score and rank, with lower scores and rank indicative of lower socioeconomic status (SES). The SEIFA score is standardized against a mean of 1000 with a standard deviation of 100, and SEIFA ranking ranged from 12 to 2450 within this cohort.

Data were analyzed using SAS software, version 9.2 (SAS Institute Inc., Cary, NC, USA). Descriptive statistics for all study variables were obtained. Univariate analyses were performed using chi-square test for equal proportion (or Fisher’s exact test where numbers were small), student’s t-test or Wilcoxon rank-sum test as appropriate, with results presented as numbers (%), mean (standard deviation) or median (interquartile range) respectively. Whilst event numbers were too small to facilitate the valid development of prediction models for outcome, to establish whether observed univariate effects were independent of patient severity, multivariate logistic and log-linear regression models were used to determine the relationships between obesity and outcome after adjustment for the APACHE III health status score, with results as odds ratios (95%CI) and geometric means (95%CI) respectively. A two sided p-value of <0.05 was considered to be statistically significant.

### Ethics Statement

Data are collected under the Quality Assurance Legislation of the Commonwealth of Australia (Part VC, Health Insurance Act 1973, Commonwealth of Australia). Such data are collected with government support and funding and are submitted on behalf of each ICU director. Each hospital allows subsequent use of this data as appropriate under the ANZICS-CORE standing procedures and in compliance with the ANZICS-CORE terms of reference.

## Results

Data was available from 173 adults admitted to ICU with laboratory-confirmed influenza during the first pandemic wave of 2009. The median age was 46 years, 55% were female, and 12.7% (or 23.2% of all females) were pregnant. Mechanical ventilation and extracorporeal membrane oxygenation (ECMO) was used in 63.6% and 6.4% of patients respectively. Median length of ICU and hospital stay was 8.4 (IQR 2.8–16.4) and 15.6 (IQR 8.0–30.5) days respectively. The overall mortality rate was 12.7%. The majority of patients were infected with either A(H1N1)pdm09 (83.8%) or seasonal influenza A/H3N2 (9.8%). A diagnosis of pneumonitis was recorded in 49.7% of patients and concurrent bacterial pneumonia was diagnosed in 13.9% of patients. Baseline patient characteristics for all patients, and those infected with A(H1N1)pdm09 (n = 145) are shown in Table 1.

**Table 1:** Baseline characteristics of patients admitted to intensive care unit during the first pandemic (H1N1) 2009 influenza wave of 2009.

Characteristic	All patients (n = 173)	Patients with A(H1N1)pdm09 only (n = 145)
Age – years		
Median	46	47
IQR	34–84	34–55
Female sex – no. (%)	95 (55%)	73 (50.3%)
Pregnant – no. (%)	22 (12.7%)	20 (13.8%)
Ethnicity – no. (%)		
Caucasian	130 (75.1%)	106 (73.1%)
Aboriginal or Torres Strait Islander	13 (7.5%)	11 (7.6%)
Maori or Pacific Islander	6 (3.5%)	6 (4.1%)
Asian	5 (2.9%)	5 (3.4%)
Other	19 (11%)	17 (11.7%)
Body mass index (kg/m^2^) – no. (%)		
<25	56 (32.4%)	45 (31%)
25–30	44 (25.4%)	38 (26.2%)
≥30	73 (42.2%)	62 (42.8%)
≥40	23 (13.3%)	20 (13.8%)
Co-morbidities – no. (%)		
Diabetes	23 (13.3%)	21 (14.5%)
Chronic lung disease	61 (35.3%)	53 (36.6%)
Chronic heart disease	26 (15%)	23 (15.9%)
APACHE II score		
Median	16	16
IQR	13–23	12–23
APACHE III score		
Median	56	55
IQR	43–73	42–73
Mechanical ventilation – no. (%)	110 (63.6%)	107 (73.8%)
ECMO – no. (%)	11 (6.4%)	11 (7.6%)
Vasopressor support – no. (%)	62 (35.8%)	52 (35.9%)
Renal replacement therapy – no. (%)	18 (10.4%)	13 (9%)
Corticosteroids – no. (%)	75 (43.4%)	63 (43.4%)
Hydrocortisone	62 (35.8%)	50 (34.5%)
Methylprednisolone	13 (7.5%)	13(9%)
Influenza subtype		
A(H1N1)pdm09	145 (83.8%)	145 (100%)
Seasonal A/H3N2	17 (9.8%)	–
Seasonal A/H1N1	1 (0.6%)	–
Untyped	10 (5.8%)	–
Influenza syndrome – no. (%)		
Viral pneumonitis/ARDS	86 (49.7%)	76 (52.4%)
Concurrent bacterial pneumonia	24 (13.9%)	17 (11.7%)
Exacerbation of airflow limitation	29 (16.7%)	23 (15.9%)
Length of stay – days		
ICU		
Median	8.38	8.6
IQR	2.8–16.4	2.8–17.9
Hospital		
Median	15.6	16.2
IQR	8.0–30.5	7.5–31.2
Death – no. (%)	22 (12.7%)	22 (15.2%)

*Definition of abbreviations*: IQR = Interquartile range; BMI = Body mass index (calculated as weight in kilograms divided by height in metres squared); APACHE = Acute Physiology, Age, and Chronic Health Evaluation; ECMO = Extracorporeal membrane oxygenation; ARDS = Acute respiratory distress syndrome; ICU = Intensive care unit.

Tables 2 and 3 show the characteristics, laboratory parameters and outcomes of non-obese, obese and morbidly obese patients admitted to ICU with severe influenza during the first pandemic wave and those with A(H1N1)pdm09 infection respectively.

**Table 2:** Characteristics, laboratory parameters and outcomes of non-obese, obese and morbidly obese patients admitted to intensive care unit during the first pandemic wave of influenza A(H1N1)pdm09.

Parameter	Non–obese (n = 100)	Obese* (n = 73)	P–value	Non–morbidly obese (n = 150)	Morbidly obese+ (n = 23)	P–value
Age (years) (Mean, SD)	48.4 (16.2)	43.4 (13.7)	0.035	47.5 (15.4)	38.6 (12.5)	0.009
Height (centimetres) (Mean, SD)	169.6 (9.0)	170.2 (9.7)	0.69	170.4 (9.0)	166.5 (10.4)	0.06
Weight (kilograms) (Mean, SD)	70.4 (13.2)	114.5 (27.1)	<0.0001	80.86 (19.8)	142.26 (28.9)	<0.0001
BMI (kg/m^2^) (Mean, SD)	24.4 (3.5)	39.7 (9.9)	<0.0001	27.7 (5.7)	51.4 (9.7)	<0.0001
APACHE II score (Median, IQR)	17 (14–23)	15 (12–19)	0.029	17 (13–23)	15 (12–19)	0.45
APACHE III score (Median, IQR)	56 (43–80)	48.5 (37–65)	0.012	55 (43–73)	41.5 (37–50)	0.012
Glasgow coma score (Mean, SD)	13.4 (3.2)	13.5 (3.3)	0.94	13.5 (3.1)	13.2 (3.7)	0.66
Temperature (^O^C) (Mean, SD)						
High	38.1 (1.3)	38.3 (1.1)	0.44	38.1 (1.2)	38.5 (1.3)	0.20
Low	36.2 (1.2)	36.5 (0.8)	0.14	36.2 (1.1)	36.8 (1.0)	0.006
Mean arterial pressure (mmHg)(Mean, SD)						
High	99.4 (17.6)	102 (15.4)	0.37	100 (16.7)	102 (17.1)	0.71
Low	63.3 (10.3)	68.8 (12.9)	0.003	65.3 (11.8)	67.8 (11.0)	0.36
Heart rate (beats/minute) (Mean, SD)						
High	114 (20.2)	109 (20.0)	0.19	113 (20.5)	106 (17.4)	0.16
Low	77.9 (15.9)	78.4 (19.3)	0.84	77.9 (17.7)	79.6 (14.7)	0.66
Respiratory rate (respirations/minute)						
High (Median, IQR)	30 (22–38)	28 (20–34)	0.09	30 (22–37)	22 (19–30)	0.007
Low (Median, IQR)	14 (12–18)	15 (12–18)	0.16	14 (12–18)	15 (12–16)	0.66
Hemoglobin (g/dL) (Median, IQR)						
High	11.9 (10.2–13.3)	12.2 (10.4–13.5)	0.26	12 (10.2–13.4)	12 (10.4–13.6)	0.69
Low	10.5 (9.0–12.0)	11.7 (9.5–12.7)	0.026	10.9 (9.0–12.3)	11.6 (9.5–12.8)	0.22
White cell (x10^9^/L) (Median, IQR)						
High	11.8 (6.4–19.3)	8.4 (6.0–12.1)	0.016	10.9 (6.2–16.2)	7.6 (5–9.5)	0.02
Low	8.5 (4.1–13)	6.2 (4.4–8.4)	0.05	7.3 (4.5–12.0)	6.4 (4.2–7.5)	0.09
Platelet count (x 10^9^/L) (Mean, SD)						
High	243 (132)	242 (118)	0.95	249 (132)	201 (65)	0.11
Low	208 (115)	208 (100)	0.98	212 (114)	183 (62)	0.28
Creatinine (µmol/L) (Median, IQR)	83 (59–146)	89.5 (66–152)	0.57	91 (66–154)	76.5 (50–100)	0.13
Urea (mmol/L) (Median, IQR)	7.6 (3.9–12.3)	6.2 (3.7–12.3)	0.45	7.9 (3.9–13.1)	4.5 (3.5–6.4)	0.029
Albumin (g/L) (Median, IQR)	26 (22–29)	29 (25.5–32.5)	0.0002	27 (23–31)	29 (27–32)	0.09
Bilirubin (µmol/L) (Median, IQR)	6 (4–10)	8 (5–13)	0.37	6.5 (4–11)	8.5 (5–11)	0.87
Glucose (mmol/L) (Mean, SD)	10.2 (5.6)	9.4 (3.4)	0.29	9.8 (5.0)	9.7 (3.7)	0.88
C-reactive protein (mg/L) (Median, IQR)						
On admission to hospital	100 (63–210)	106 (49–189)	0.71	115 (62–210)	67 (44–140)	0.08
On admission to ICU	113 (65–225)	110 (65–190)	0.47	112 (67–213)	87 (51–200)	0.39
pH (Mean, SD)	7.3 (0.2)	7.3 (0.1)	0.10	7.3 (0.2)	7.4 (0.1)	0.19
HCO_3_ (mmol/L) (Mean, SD)	21.8 (7.0)	24.5 (6.2)	0.013	22.2 (6.6)	27.6 (6.2)	0.0004
P_a_O_2_ (mmHg) (Median, IQR)	75 (63–95)	70 (60–90)	0.26	75 (63–95)	67 (50–79)	0.034
F_I_O_2_ (mmHg) (Mean, SD)	0.7 (0.3)	0.8 (0.3)	0.13	0.7 (0.3)	0.9 (0.2)	0.002
P_a_CO_2_ (mmHg) (Median, IQR)	41 (34–49)	44 (37–54)	0.08	44.1 (14.8)	54.1 (18.8)	0.006
Influenza subtype – no. (%)						
Pandemic (H1N1) 2009	83 (83%)	62 (85%)	0.84	125 (83%)	20 (87%)	1.00
Seasonal A/H3N2	11 (11%)	6 (8%)	0.61	15 (10%)	2 (9%)	1.00
Seasonal A/H1N1	1 (1%)	0	1.00	1 (1%)	0	1.00
Untyped	5 (5%)	5 (7%)	0.74	9 (6%)	1 (4%)	1.00
Influenza syndrome – no. (%)						
Viral pneumonitis/ARDS	43 (44%)	43 (61%)	0.029	71 (49%)	15 (68%)	0.09
Concurrent bacterial pneumonia	15 (15%)	9 (13%)	0.64	21 (14%)	3 (14%)	0.92
Exacerbation of airflow limitation	17 (18%)	12 (17%)	0.95	26 (18%)	3 (14%)	0.62
Mechanical ventilation – no. (%)	64 (64%)	46 (63%)	1.00	92 (61%)	18 (78%)	0.16
Duration of mechanical ventilation(days) (Median, IQR)	5 (0–11)	8 (1–7)	0.06	5 (0–12)	9.5 (3–14)	0.18
ECMO – no. (%)	4 (4%)	7 (10%)	0.21	7 (5%)	4 (17%)	0.04
Vasopressor – no. (%)	36 (42%)	26 (42%)	0.96	53 (42%)	9 (45%)	0.78
Renal replacement therapy – no. (%)	10 (11%)	8 (13%)	0.79	16 (12%)	2 (10%)	0.76
Length of stay (days) (Median, IQR)						
ICU	6.8 (2.5–13.0)	10.2 (3.0–20.4)	0.13	7.8 (2.5–14.9)	11.2 (4.5–19.2)	0.15
Excluding those that died	6.8 (3.7–12.3)	11.9 (3.9–23.3)	0.017	8.9 (3.7–15.2)	12.1 (8.5–19.8)	0.07
Hospital	15.1 (7.6–27.3)	17.2 (8.3–32.3)	0.43	15.4 (7.5–30.4)	17.2 (8.3–35.2)	0.61
Death – no. (%)						
In ICU	15 (17%)	3 (5%)	0.02	16 (12%)	2 (10%)	0.74
In hospital	18 (20%)	4 (6%)	0.016	20 (15%)	2 (9%)	0.48
Median household income/week(AUD) (Mean, SD)	1075 (264)	1012 (210)	0.10	1052 (249)	1026 (209)	0.64
SEIFA score (Mean, SD)	978 (80)	976 (70)	0.86	978 (78)	971 (64)	0.67
SEIFA rank (Mean, SD)	1099 (725)	1060 (666)	0.72	1105 (695)	934 (723)	0.28

*Definitions of abbreviations*: APACHE = Acute Physiology, Age, and Chronic Health Evaluation; ARDS = Acute respiratory distress syndrome; AUD = Australian dollar; BMI = Body mass index (calculated as weight in kilograms divided by height in metres squared); ECMO = Extracorporeal membrane oxygenation; ICU = Intensive care unit; IQR = Interquartile range; SD = Standard deviation; SEIFA = Socioeconomic indexes for areas.

* Obese BMI ≥30 kg/m^2^.

+ Morbidly obese BMI ≥40 kg/m^2^.

**Table 3:** Characteristics, laboratory parameters and outcomes of non-obese, obese and morbidly obese patients with influenza A(H1N1)pdm09 admitted to intensive care unit during the first pandemic wave.

Parameter	Non–obese (n = 83)	Obese* (n = 62)	P–value	Non–morbidly obese (n = 125)	Morbidly obese+ (n = 20)	P–value
Age (years) (Mean, SD)	48 (16.1)	43.1 (13.5)	0.05	46.9 (15.3)	39.5 (13.2)	0.04
Height (centimetres) (Mean, SD)	170 (8.8)	170.2 (10.3)	0.87	170.7 (9.1)	166 (11.1)	0.038
Weight (kilograms) (Mean, SD)	70.9 (12.4)	116.3 (28.3)	<0.0001	81.7 (19.9)	144.5 (30.3)	<0.0001
BMI (kg/m^2^) (Mean, SD)	24.5 (3.4)	40.4 (10.3)	<0.0001	27.9 (5.8)	52.5 (9.9)	<0.0001
APACHE II score (Median, IQR)	17 (14–23)	15 (11–18)	0.018	17 (12–23)	14.5 (12–18)	0.58
APACHE III score (Median, IQR)	58 (49–81)	48 (37–61)	0.009	58 (45–76)	41 (37–48)	0.005
Glasgow coma score (Mean, SD)	13.4 (3.3)	13.6 (3.2)	0.7	13.5 (3.3)	13.5 (3.1)	0.93
Temperature (^O^C) (Mean, SD)						
High	38.1 (1.4)	38.3 (1.2)	0.3	38.2 (1.2)	38.5 (1.4)	0.23
Low	36.2 (1.3)	36.5 (0.9)	0.09	36.2 (1.1)	36.8 (1.0)	0.01
Mean arterial pressure (mmHg)(Mean, SD)						
High	99.7 (18.4)	101.1 (14.5)	0.63	100.3 (17.2)	100.4 (14.4)	0.98
Low	63.2 (10.5)	69.4 (13.2)	0.003	65.3 (12.2)	69 (11.2)	0.22
Heart rate (beats/minute) (Mean, SD)						
High	113.8 (20.2)	107.3 (19.9)	0.05	112.3 (20.6)	103.5 (16.3)	0.08
Low	77.3 (15)	77.3 (18.2)	0.99	77.2 (16.8)	78 (14)	0.85
Respiratory rate (respirations/minute)						
High (Mean, SD)	31.6 (12.1)	29 (11.6)	0.22	31.5 (12.2)	24.3 (7)	0.015
Low (Mean, SD)	14.8 (3.9)	16.6 (8.7)	0.1	15.6 (6.7)	15 (3.6)	0.70
Hematocrit (Mean, SD)						
High	0.4 (0.1)	0.4 (0.1)	0.21	0.4 (0.1)	0.4 (0.1)	0.013
Low	0.3 (0.1)	0.4 (0.1)	0.01	0.3 (0.1)	0.4 (0.1)	0.001
White cell (x10^9^/L) (Median, IQR)						
High	11.4 (6.4–16.7)	8.2 (6–11.3)	0.035	10 (6.2–16.1)	7.9 (6–9.5)	0.06
Low	7.3 (4.3–12.3)	6.2 (4.4–8.3)	0.12	6.9 (4.4–11.4)	6.5 (4.4–7.5)	0.2
Platelet count (x 10^9^/L) (Mean, SD)						
High	227.4 (121.8)	225.7 (97.9)	0.93	230.6 (116.7)	201.5 (69.7)	0.32
Low	192.8 (105.9)	198 (84.9)	0.77	197 (101.3)	183.5 (67.2)	0.6
Creatinine (µmol/L) (Median, IQR)	83 (59–148)	89.5 (67.5–166)	0.68	88.5 (66–165)	79 (49–100)	0.15
Urea (mmol/L) (Median, IQR)	7.6 (3.9–13.3)	5.8 (3.5–10.8)	0.37	7.7 (3.9–13.3)	4.5 (3.4–7.8)	0.06
Albumin (g/L) (Median, IQR)	26 (23–29)	29 (25–32)	0.006	27 (24–30.5)	29 (27–31)	0.09
Bilirubin (µmol/L) (Median, IQR)	6 (5–10)	7 (5–12)	0.8	7 (5–12)	8 (3–11)	0.71
Glucose (mmol/L) (Mean, SD)	10.4 (6)	9.5 (3.6)	0.29	10 (5.3)	9.9 (3.8)	0.97
C-reactive protein (mg/L) (Median, IQR)						
On admission to hospital	98.5 (56–210)	110 (49–188)	0.86	117.5 (57–209)	68 (39–140)	0.08
On admission to ICU	110 (55–216)	112.5 (65–188)	0.8	110 (63–210)	100.8 (52.5–193)	0.53
pH (Mean, SD)	7.29 (0.2)	7.4 (0.1)	0.024	7.3 (0.2)	7.4 (0.1)	0.05
HCO_3_ (mmol/L) (Mean, SD)	21.5 (7.2)	24.6 (6.5)	0.013	22 (6.8)	28.1 (6.3)	0.0004
P_a_O_2_ (mmHg) (Median, IQR)	73 (64–97)	68.5 (60–89)	0.25	72 (63–102)	68 (50–79)	0.11
F_I_O_2_ (mmHg) (Mean, SD)	0.7 (0.3)	0.8 (0.3)	0.13	0.7 (0.3)	0.9 (0.2)	0.002
P_a_CO_2_ (mmHg) (Median, IQR)	41 (34–48)	44 (37–54)	0.37	41 (34–49)	48 (42–55)	0.016
Influenza syndrome – no. (%)						
Viral pneumonitis/ARDS	38 (46%)	38 (62%)	0.07	63 (50%)	13 (65%)	0.24
Concurrent bacterial pneumonia	10 (12%)	7 (11%)	1.00	15 (12%)	2 (10%)	1.00
Exacerbation of airflow limitation	13 (16%)	10 (16%)	1.00	20 (16%)	3 (15%)	1.00
Mechanical ventilation – no. (%)	53 (64%)	39 (63%)	1.00	76 (61%)	16 (80%)	0.13
Duration of mechanical ventilation(days) (Median, IQR)	5 (0–11)	8 (1–7)	0.06	5 (0–12)	9.5 (3–14)	0.18
ECMO – no. (%)	4 (5%)	7 (11%)	0.20	7 (6%)	4 (20%)	0.046
Vasopressor – no. (%)	33 (40%)	19 (31%)	0.3	45 (36%)	7 (4%)	1.00
Renal replacement therapy – no. (%)	8 (10%)	5 (8%)	1.00	12 (10%)	1 (5%)	1.00
Length of stay (days) (Median, IQR)						
ICU	7.24 (2.5–13.1)	11.2 (3.1–23.1)	0.08	7.9 (2.5–16.6)	11.5 (6.8–23.7)	0.06
Excluding those that died	7.1 (3.4–12.7)	12.5 (3.9–24)	0.02	9 (3.4–17.2)	12.5 (10.3–19.8)	0.07
Hospital	15.4 (7.3–27.2)	19.4 (8–34.2)	0.25	15.6 (6.7–30.4)	18.4 (11.6–35.2)	0.28
Death – no. (%)						
In ICU	16 (19%)	5 (8%)	0.09	19 (15%)	2 (10%)	0.74
In hospital	18 (22%)	4 (6%)	0.018	20 (16%)	2 (10%)	0.74
Median household income/week(AUD) (Mean, SD)	1065.8 (262.5)	1004.8 (215.2)	0.14	1040.8 (249.4)	1033.2 (216.6)	0.9
SEIFA score (Mean, SD)	975.9 (82.3)	974.6 (73.4)	0.92	976.4 (80.2)	968.7 (67.1)	0.68
SEIFA rank (Mean, SD)	1094 (720.3)	1066.4 (676.7)	0.82	1101.2 (695.7)	963.2 (731.3)	0.41

*Definitions of abbreviations*: APACHE = Acute Physiology, Age, and Chronic Health Evaluation; ARDS = Acute respiratory distress syndrome; AUD = Australian dollar; BMI = Body mass index (calculated as weight in kilograms divided by height in metres squared); ECMO = Extracorporeal membrane oxygenation; ICU = Intensive care unit; IQR = Interquartile range; SD = Standard deviation; SEIFA = Socioeconomic indexes for areas.

* Obese BMI ≥30 kg/m^2^.

+ Morbidly obese BMI ≥40 kg/m^2^.

Of 173 patients, 100 (57.8%), 73 (42.2%) and 23 (13.3%) had BMI <30 kg/m^2^, ≥30 kg/m^2^ and ≥40 kg/m^2^ respectively. On univariate analyses, obese patients (BMI ≥30 kg/m^2^) were younger (mean age 43.4 vs. 48.4 years, p = 0.035) and more likely to be diagnosed with pneumonitis (61% vs. 44%, p = 0.029) when compared to non-obese patients. Morbidly obese patients (BMI ≥40 kg/m^2^) were more likely to require ECMO (17.4% vs. 4.7%, p = 0.04). These trends were also observed when patients with A(H1N1)pdm09 only were analyzed.

While obesity was significantly more prevalent amongst patients with pneumonitis (50% vs. 33% p = 0.029), this result was no longer significant after adjustment for patient severity (OR 1.75 [0.91–3.37], p = 0.10). However, obesity (OR 2.19 [1.12–4.71], p = 0.045), but not morbid obesity (OR 2.11 ([0.69–6.45], p = 0.18), was an independent predictor of pneumonitis after adjusting for age and chronic lung disease.

ICU and hospital mortality rates were significantly higher for non-obese patients (17% vs. 5%, p = 0.02 and 20% vs. 6%, p = 0.016 respectively), but median APACHE II and APACHE III scores on admission were also significantly higher (17 vs. 15, p = 0.029 and 56 vs. 48.5, p = 0.012). Consequently, after adjustment for patient severity, obesity was no longer a significant predictor of either ICU or hospital mortality (OR 0.42 [0.10–1.77], p = 0.24 and OR 0.37 [0.11–1.30)], p = 0.12 respectively).

Similarly, mortality rates between morbidly obese and non-morbidly obese patients were significant at a univariate level, but became non-significant after adjustment for patient severity. There was a trend towards longer ICU and hospital length of stay (LOS) in obese and morbidly obese patients. However, for ICU survivors, obese patients did have longer median ICU LOS (11.9 vs. 6.8 days, p = 0.017), with this result remaining significant after adjustment for patient severity (geometric mean (95%CI) 9.4 [7.1–12.4] vs. 6.1 [4.8–7.9], p = 0.03).

CRP levels were not significantly higher when obese and non-obese patients were compared on admission to hospital and ICU. Obese and morbidly obese patients tended to have lower median household incomes, SEIFA scores, and SEIFA rankings.

## Discussion

Although obese patients are more likely to be hospitalized with A(H1N1)pdm09 infection [18], [21], there are conflicting data on whether obesity is an independent risk factor for increased mortality in severe A(H1N1)pdm09 infection. Díaz *et al*, Kumar *et al* and Estenssoro *et al* all noted that obese patients were more likely to require mechanical and prolonged ventilation, but not more likely to die [22], [23], [24]. Rodríguez *et al* in fact noted improved mortality rates of obese patients with severe A(H1N1)pdm09 infection, paralleling the experience of obese patients suffering other critical illness [25], [26], [27]. This is in contrast to data suggesting that obesity is an independent risk factor for increased mortality [6], [17], [18], [19], [20]. A potential explanation for this discrepancy is that only subsets of obese patients are at increased risk for severe infection; obese patients that develop pneumonitis.

Similar to previous reports [28], obese and morbidly obese patients in our cohort were more likely to develop pneumonitis compared to non-obese patients. Although Rodríguez *et al* noted that obese patients had improved mortality rates on univariate analyses, logistic regression analysis showed that higher APACHE II scores were independently associated with increased mortality [25]. Our findings are concordant with those of Rodríguez *et al*; non-obese patients had higher mortality rates on univariate analysis, but this observation was not maintained after adjustment for severity of illness as reflected by APACHE II and APACHE III scores. On the other hand, obese patients may have had less severe disease as reflected by higher albumin levels and lower white cell counts, CRP, urea, APACHE II and APACHE III scores.

Previous meta-analyses prior to the 2009 pandemic have identified that obese patients have similar or greater levels of morbidity, but not mortality, following admission to ICU and that obesity may in fact be protective [26], [29]. Our data suggests that both obese and non-obese patients have similar levels of mortality; however, non-obese patients tended to be older. Although younger patients with A(H1N1)pdm09 are more likely to be hospitalized, ICU admission and mortality rates were higher in the elderly. Older patients are more likely to have co-morbid conditions, although younger patients were more likely to be obese and pregnant [17], [30].

In the present study, obesity was an independent predictor of pneumonitis after adjusting for age and chronic lung disease. Furthermore, clinicians may have intervened with more advanced levels of respiratory support in the obese patients pre-emptively and more readily prior to even more significant respiratory failure. Although our obese patients were more likely to develop pneumonitis, they were also more likely to recover once the acute lung insult resolved. The duration of mechanical ventilation was similar between obese and non-obese patients, comparable to the experience of critically ill patients with respiratory failure prior to the 2009 pandemic [26].

Obese and morbidly obese patients had greater levels of hypoxemia, as reflected by the lower partial pressures of arterial oxygen and higher fractions of inspired oxygen. Presumably, obesity *per se* results in greater levels of hypoxemia for any given pulmonary insult, as obese patients have reduced lung and chest wall compliance, and higher airway resistance, resulting in greater ventilation/perfusion mismatching [26]. There was an increased use of ECMO in both these groups of patients, reflecting perceived inadequate ventilation of these patients by conventional mechanical ventilation. There is conflicting evidence on the efficacy of ECMO in this setting; Noah *et al* observed improved mortality rates [31], whilst Davies *et al* noted worse outcomes in patients receiving ECMO for severe A(H1N1)pdm09 infection compared to those receiving mechanical ventilation alone [32]. However, in the latter group, those receiving ECMO had severe respiratory failure treated with at least one other method of rescue ARDS therapy (recruitment manoeuvres, prone-positioning, high-frequency oscillatory ventilation and/or inhaled nitric oxide or prostacyclin) prior to ECMO, and the majority were referred from other hospitals [32].

Previous investigators have observed significantly higher levels of pro- and anti-inflammatory cytokines (including IL-6, IL-8, MCP-1) in patients with severe A(H1N1)pdm09 infection [33], [34], [35]. In contrast to data from Hagau *et al* [35], our data suggests that CRP alone may not be an accurate marker for excess cytokine production in severe A(H1N1)pdm09 infection, as levels were similar in both obese and morbidly obese, compared to non-obese, patients on admission to hospital and ICU, despite the increased frequency of pneumonitis in the obese group. A plausible explanation for similar CRP levels in both groups of patients is the possibility of concurrent bacterial co-infection, as other investigators have noted higher CRP values in this setting [19], [36]. Although evidence of bacterial pneumonia was present in only 13.9% of patients, this may have been underestimated as radiological evidence and microbiological confirmation of bacterial pneumonia was not always available. Other investigators have suggested co-infection rates of ∼25% [23], [37].

The prevalence of obesity is inversely proportional to the SES in children and adults [38], [39]. In addition, people from lower socioeconomic backgrounds have increased rates of influenza, and are less likely to be vaccinated against influenza [40], [41]. Although not statistically significant, trends of lower SEIFA scores, SEIFA rankings and median household incomes, based on residential postcode areas, were observed in our obese patients. However, postcodes areas may contain a mix of people from low, medium or high socioeconomic backgrounds, potentially confounding the use of postcodes alone as a measure of SES.

Unlike some countries in the Northern Hemisphere, Australia did not have a “pre-first” pandemic wave, thus providing us with a unique opportunity to study the effects of a newly circulating pandemic influenza virus on susceptible obese populations with little pre-existing immunity. The data presented herein was collected from nine different ICUs with ECMO facilities during the first pandemic wave in the Southern Hemisphere, which coincided with the winter of 2009, and prior to the introduction of the monovalent A(H1N1)pdm09 vaccine in Australia [42].

BMI and CRP data were not available for the entire cohort of patients admitted to Australian ICUs during the first pandemic wave [1]. Nevertheless, the 173 patients in our study are representative of the original cohort, with similar rates of obesity, demographics, and outcomes. Although CRP data were obtained retrospectively, this parameter was measured at the time of hospital and ICU admission. Influenza A subtypes were also not available in all patients, as retrospective diagnoses were made serologically in some instances [43]. However, these were likely to be A(H1N1)pdm09, given the epidemiology of circulating viruses at the time. We were also not able to retrospectively measure levels of pro- and anti-inflammatory cytokines or influenza viral loads (and hence viral clearance), which we postulate would have been higher in obese patients. The use of anti-inflammatory and/or immunomodulatory agents such as corticosteroids and statins [44], [45], [46], [47] that may mitigate the excessive inflammatory response were also not recorded.

In conclusion, obese patients with severe A(H1N1)pdm09 infection from the first pandemic wave in Australia were more likely to develop pneumonitis compared to non-obese patients, but mortality rates were similar between the two groups after adjusting for severity of disease. Although there is on-going debate as to whether obesity is a risk factor for severe A(H1N1)pdm09 infection, annual influenza vaccination should be prioritized in this group given the increased risk of serious complications from seasonal influenza infection [6], [8], [21]. Current Australian and United Kingdom vaccination guidelines do not specifically recommend annual influenza vaccination in the obese [48], [49], in contrast to guidelines in the USA [50]. Further studies evaluating accurate markers of pneumonitis should also be pursued.
